# Supplementary material for: Early life experience sets hard limits on motor learning as evidenced from artificial arm use
Source: eLife. 2021 Oct 4;10:e66320. doi: 10.7554/eLife.66320 (PMC8523152; doi:10.7554/eLife.66320)
Supplement: Supplementary file 1. — (a) Main analysis while controlling for artificial arm/nondominant arm side. Results of a follow-up ANCOVA analysis showing no effects of artificial arm side (L vs. R) on artificial arm reaching errors. Our main finding of a significant group effect was also unaffected by accounting for the side of the arm making the reaches. (b) Main analysis while controlling for residual limb length. Results of a follow-up ANCOVA analysis showing no effects of residual limb length on artificial arm reaching errors. Our main finding of a significant group effect was also unaffected by accounting for residual limb length. Note that this analysis only includes artificial arm users (congenital and acquired) as controls have a complete arm and therefore no residual limb length (c) Comparing artificial arm error noise while controlling for artificial arm bias. Results of a follow-up ANCOVA analysis showing that while there is a significant relationship between bias and noise, the group differences in error noise are independent of bias. (d) Analysis of reaching errors comparing the main task and the 2D localization task. Participants made overall larger errors in the 2D localization task compared to the main task which included visual feedback. (e) Analysis of movement time comparing the main task and the 2D localization task. Participants took longer to move to the target in the 2D localization task compared to the main task which included visual feedback. [file elife-66320-supp1.docx]

# Supplementary full statistical reports

| **ANCOVA – Dependent variable: Artificial arm absolute errors** | | | | | | | | | | |  |
| --- | --- | --- | --- | --- | --- | --- | --- | --- | --- | --- | --- |
| **Factors** | | **SS** | | **df** | | **MS** | | **F** | | **p** |  |
| Group – Fixed factor |  | 0.719 |  | 2 |  | 0.360 |  | 12.117 |  | < .001 |  |
| Artificial arm side – Fixed factor |  | 0.033 |  | 1 |  | 0.033 |  | 1.119 |  | 0.296 |  |
| Intact-arm absolute errors - Covariate |  | 0.835 |  | 1 |  | 0.835 |  | 28.127 |  | < .001 |  |
| Group ✻ Artificial arm side interaction |  | 0.013 |  | 2 |  | 0.006 |  | 0.216 |  | 0.807 |  |
| Residuals |  | 1.306 |  | 44 |  | 0.030 |  |  |  |  |  |
|  | | | | | | | | | | |  |
|  | | | | | | | | | | |  |

*Supplementary File 1a.* ***Main analysis while controlling for artificial arm/nondominant-arm side.*** Results of a follow-up ANCOVA analysis showing no effects of artificial arm side (L vs R) on artificial arm reaching errors. Our main finding of a significant group effect was also unaffected by accounting for the side of the arm making the reaches.

| **ANCOVA – Dependent variable: Artificial arm absolute errors** | | | | | | | | | | |  |
| --- | --- | --- | --- | --- | --- | --- | --- | --- | --- | --- | --- |
| **Factors** | | **SS** | | **df** | | **MS** | | **F** | | **p** |  |
| Group – Fixed factor |  | 0.137 |  | 1 |  | 0.137 |  | 5.065 |  | 0.032 |  |
| Residual-limb length- Covariate |  | 0.042 |  | 1 |  | 0.042 |  | 1.565 |  | 0.221 |  |
| Intact-arm absolute errors - Covariate |  | 0.318 |  | 1 |  | 0.318 |  | 11.768 |  | 0.002 |  |
| Residuals |  | 0.758 |  | 28 |  | 0.027 |  |  |  |  |  |
|  | | | | | | | | | | |  |
|  | | | | | | | | | | |  |

*Supplementary File 1b.* ***Main analysis while controlling for residual-limb length.*** Results of a follow-up ANCOVA analysis showing no effects of residual-limb length on artificial arm reaching errors. Our main finding of a significant group effect was also unaffected by accounting for residual-limb length. Note that this analysis only includes artificial arm users (congenital and acquired) as controls have a complete arm and therefore no residual-limb length.

| **ANCOVA – Dependent variable: Artificial arm error noise** | | | | | | | | | | |  |
| --- | --- | --- | --- | --- | --- | --- | --- | --- | --- | --- | --- |
| **Factors** | | **SS** | | **df** | | **MS** | | **F** | | **p** |  |
| Group – Fixed factor |  | 1.434 |  | 2 |  | 0.717 |  | 12.405 |  | < .001 |  |
| Artificial arm bias - Covariate |  | 0.404 |  | 1 |  | 0.404 |  | 6.991 |  | 0.011 |  |
| Intact-arm noise - Covariate |  | 0.460 |  | 1 |  | 0.460 |  | 7.962 |  | 0.007 |  |
| Residuals |  | 2.659 |  | 46 |  | 0.058 |  |  |  |  |  |
|  | | | | | | | | | | |  |
|  | | | | | | | | | | |  |

*Supplementary File 1c.* ***Comparing artificial arm error noise while controlling for artificial arm bias.*** Results of a follow-up ANCOVA analysis showing that while there is a significant relationship between bias and noise, the group differences in error noise are independent of bias.

| **Repeated Measures ANOVA** | | | | | | | | | | | | | | | | | | | | |  |  |
| --- | --- | --- | --- | --- | --- | --- | --- | --- | --- | --- | --- | --- | --- | --- | --- | --- | --- | --- | --- | --- | --- | --- |
| **Within Subjects Effects** | | | **SS** | | | **df** | | **MS** | | | | | **F** | | | | **p** | | **η² _p_** | |  |  |
| Task |  | | 46.846 | |  | 1 |  | 46.846 | | | |  | 394.376 | | |  | < .001 |  | 0.898 |  |  |  |
| Task ✻ Group |  | | 0.191 | |  | 2 |  | 0.095 | | | |  | 0.802 | | |  | 0.455 |  | 0.034 |  |  |  |
| Residuals |  | | 5.345 | |  | 45 |  | 0.119 | | | |  |  | | |  |  |  |  |  |  |  |
| Hand |  | | 2.102 | |  | 1 |  | 2.102 | | | |  | 29.387 | | |  | < .001 |  | 0.395 |  |  |  |
| Hand ✻ group |  | | 0.199 | |  | 2 |  | 0.099 | | | |  | 1.390 | | |  | 0.260 |  | 0.058 |  |  |  |
| Residuals |  | | 3.218 | |  | 45 |  | 0.072 | | | |  |  | | |  |  |  |  |  |  |  |
| Hand ✻ Task |  | | 0.434 | |  | 1 |  | 0.434 | | | |  | 9.922 | | |  | 0.003 |  | 0.181 |  |  |  |
| Hand ✻ Task ✻ Group |  | | 0.455 | |  | 2 |  | 0.227 | | | |  | 5.194 | | |  | 0.009 |  | 0.188 |  |  |  |
| Residuals |  | | 3.218 | |  | 45 |  | 0.072 | | | |  |  | | |  |  |  |  |  |  |  |
|  | | | | | | | | | | | | | | | | | | | | |  |  |
|  | | | | | | | | | | | | | | | | | | | | | |  |
| **Between Subjects Effects** | | **SS** | | | | | **df** | | | **MS** | | | | **F** | | | **p** | **η² _p_** | | | |  |
| Group | |  | | 0.728 | |  | 2 | |  | | 0.364 | |  | | 2.911 |  | 0.065 |  | 0.115 | |  | |
| Residuals | |  | | 5.631 | |  | 45 | |  | | 0.125 | |  | |  |  |  |  |  | |  | |
|  | | | | | | | | | | | | | | | | | | | | | |  |
| Note.  Type III Sum of Squares  *Supplementary File 1d.* **Analysis** **of reaching errors comparing the main task and the 2D localisation task*.*** Participants made overall larger errors in the 2D localisation task compared to the main task which included visual feedback. | | | | | | | | | | | | | | | | | | | | | |  |

| **Repeated Measures ANOVA** | | | | | | | | | | | | | |
| --- | --- | --- | --- | --- | --- | --- | --- | --- | --- | --- | --- | --- | --- |
| **Within Subjects Effects** | | **SS** | | **df** | | **MS** | | **F** | | **p** | | **η² _p_** | |
| Task |  | 6.294e +6 |  | 1 |  | 6.294e +6 |  | 56.625 |  | < .001 |  | 0.557 |  |
| Task ✻ Group |  | 73281.726 |  | 2 |  | 36640.863 |  | 0.330 |  | 0.721 |  | 0.014 |  |
| Residuals |  | 5.002e +6 |  | 45 |  | 111148.771 |  |  |  |  |  |  |  |
| Hand |  | 393511.134 |  | 1 |  | 393511.134 |  | 42.156 |  | < .001 |  | 0.484 |  |
| Hand ✻ Group |  | 57099.163 |  | 2 |  | 28549.581 |  | 3.058 |  | 0.057 |  | 0.120 |  |
| Residuals |  | 420063.441 |  | 45 |  | 9334.743 |  |  |  |  |  |  |  |
| Hand ✻ Task |  | 63397.897 |  | 1 |  | 63397.897 |  | 6.514 |  | 0.014 |  | 0.126 |  |
| Hand ✻ Task ✻ Group |  | 48824.281 |  | 2 |  | 24412.140 |  | 2.508 |  | 0.093 |  | 0.100 |  |
| Residuals |  | 420063.441 |  | 45 |  | 9334.743 |  |  |  |  |  |  |  |
|  | | | | | | | | | | | | | |

| **Between Subjects Effects** | | | | | | | | | | | | | |
| --- | --- | --- | --- | --- | --- | --- | --- | --- | --- | --- | --- | --- | --- |
| **Cases** | | **Sum of Squares** | | **df** | | **Mean Square** | | **F** | | **p** | | **η² _p_** | |
| Group |  | 319359.770 |  | 2 |  | 159679.885 |  | 0.978 |  | 0.384 |  | 0.042 |  |
| Residuals |  | 7.349e +6 |  | 45 |  | 163305.004 |  |  |  |  |  |  |  |
|  | | | | | | | | | | | | | |
| Note.  Type III Sum of Squares | | | | | | | | | | | | | |

*Supplementary File 1e.* **Analysis** **of movement time comparing the main task and the 2D localisation task*.*** Participants took longer to move to the target in the 2D localisation task compared to the main task which included visual feedback.
